# Reduction of a Dislocation of Both Bones of the Forearm Backwards, Five Months after the Accident—With Remarks

**Published:** 1866-04

**Authors:** W. F. Westmoreland

**Affiliations:** Prof. of Surgery in the Atlanta Medical College


*Reduction of a Dislocation of both Bones of the Forearm backwards, five months after the accident—with remarks.*—By W. F. WESTMORELAND, M. D., Prof. of Surgery in the Atlanta Medical College.

Mrs. D———, of Cherokee county, Georgia, twenty-two years of age, medium size, well developed muscular system, consulted me on the 1st of March, 1866, for an injury of the elbow-joint received five months previous.

She gave the following history of the case: On the 2nd of October, 1865, she was thrown from a buggy, and in the fall received an injury to the elbow-joint. A physician was called to see her soon after the accident, and assured her that there was neither a dislocation nor fracture, and that she need have no fears in regard to any serious results. Not regaining the use of the limb, in six weeks or two months after the injury, she consulted another physician in the vicinity, who told her that she had a displacement of the bones of the elbow-joint. He placed her under the influence of chloroform, and attempted the reduction, but after repeated efforts, failed to reduce the dislocation.

Upon examination, I found a dislocation of the ulna and radius backwards; the arm almost straight, with not the least motion of the elbow-joint. The articular surface of the humerus was resting upon the anterior surface of the bones of the forearm; the internal condyle was readily detected, an inch to an inch and a half below and anterior to the olecranon process; the coronoid process of the ulna evidently resting in the olecranon fossa.

After giving her, in detail, the difficulties and dangers of an attempt at reduction, with the many chances of a complete failure, she did not hesitate to decide to have the effort made.

On the 2d of March, just five months after the accident, assisted by Drs. D'Alvigny, Crawford, Orme, Ray and Westbrook, I succeeded, in the following manner, and without any great difficulty, in reducing the dislocation:

The patient was placed upon the edge of the bed, partially turned upon the side corresponding with the injured arm, with the shoulders slightly elevated by means of pillows.

When she was fully under the influence of chloroform, I seated myself upon the edge of the bed, and with my knee in the bend of the arm, forcibly, but slowly, flexed the arm to near a right angle, thus breaking up the new attachments, and putting upon the strech, and to a certain extent, elongating the triceps muscle. After several times forcibly flexing the arm in this way, one assistant grasped the forearm with me, while another placed his forearm in the bend of the elbow, so as to act more directly upon the articular surface of the humerus than could be done with the knee; the knee, however, was kept in the same position, and not only assisted the arm of my assistant in making counter-extension, but by its contact with the bones of the forearm when the arm was flexed, acted as a fulcrum to raise the coronoid process out of the olecranon fossa and over the articular surface of the humerus. Extension was now made, commencing with the arm flexed to an angle of about forty-five degrees, and as the extension was continued, gradually approached a right angle. The extension was continuous, and the force gradually increased. In a very few minutes, it was evident that the soft parts were yielding, and that the bones were changing their position. In less than twenty minutes after extension was commenced, the reduction was accomplished. The forearm was now flexed at a right angle, and secured in that position by means of adhesive plaster passed from the forearm to the shoulder and to the arm.

A decided dose of sulph. morphine was administered, to be repeated every few hours if the pain, which immediately after the reduction was excruciating, continued. The application of cold to the elbow-joint was continuous. The pain continued for twenty-four hours. The tumefaction at the expiration of forty-eight hours was considerable—extending from the wrist to the shoulder. The pulsation in the radial artery, immediately after the reduction, was barely perceptible, but gradually increased in fullness until, at the expiration of three or four days, it differed but little, if any, from the radial artery of the opposite side. The functions of the ulnar nerve were also considerably interfered with. For several days there was a complete paralysis of the little finger, and partial paralysis of the ring and middle finger. The sixth day after the reduction, the tenderness and tumefaction had, to my surprise, subsided to a considerable extent. On the seventh day passive motion of the elbow-joint was commenced and continued once a day.

The day she left for her home—the fourteenth after the reduction—the tenderness, tumefaction and paralysis had greatly subsided, and she was able to slightly flex and extend the forearm.

Was the attempt at reduction in this case justifiable? As it terminated, the patient was greatly benefitted, but had an accident occurred, resulting in the loss of the limb or the life of the patient, what would have been the verdict of the profession? In other words, are we justifiable in attempting the reduction of a dislocation of the elbow-joint five months after the displacement?

In my judgement the attempt should always be made where there are no marked contraindications to the procedure. The standard authors, however, of the present day, as will be seen from the following extracts, do not justify the effort. M. Vidal, in his work on *External Pathology*, in discussing neglected dislocations of the elbow-joint, says:

"According to Boyer it is rarely possible, after a month or six weeks, to reduce this dislocation. A. Berard mentions a case reduced by Desault two months after the accident. M. Malgaigne, and M. Lisfranc, is said to have reduced an incomplete dislocation after near four months."

Prof. Gross, in his *System of Surgery*, says: "It has long been a question with surgeons at what period, after the occurrence of a dislocation, it should be considered as impracticable to effect reduction. The question, as might have been expected, has been differently answered by different observers, and by the same observers for different joints. Thus, Sir Astley Cooper, who has always been regarded as the leading authority upon the subject, thought that three months for the shoulder, and eight weeks for the hip, might be set down as the limit beyond which any efforts of this kind, except in persons of very lax or advanced age, would be highly imprudent—an opinion which accords so well with general experience as, in my judgment, to entitle it to be considered as a law. It cannot be denied that the law has exceptions, but this only serves the more fully to establish its validity. Thus, in relation to at least one of the joints in question, quite a number of cases have been reported of reduction, at from four to seven months after the receipt of the injury." He further says: "For the ginglymoid articulations the period is still more limited, although, in this respect, it varies a good deal among themselves. In relation to the elbow-joint, which is the best type of the ginglymoid class, I have found, in quite a considerable number of cases, that any attempt at reduction, however perseveringly or judiciously continued, will generally prove completely abortive after the third week."

M. Nelaton, in his most excellent work, *Pathologie Chrurgicale*, in speaking of dislocations backwards of the bones of the forearm, says: "In a very short time they become irreducible. Still Boyer succeeded in reducing one six weeks after the accident in a child ten years old. A. Cooper reduced one several weeks after the accident; Desault succeeded in one case at the end of two months. M. Malgaigne mentions a case reduced by M. Lisfranc, at the end of four months—he regarded it as incomplete."

From Prof. Hamilton's work on *Fractures and Dislocations*, we make the following extract. In speaking of dislocations of the elbow-joint, he says: "If much time has elapsed since the occurrence of the dislocation the reduction is accomplished with difficulty, if, indeed, it can be reduced at all. There are many cases upon record, however, in which surgeons have been successful after the lapse of many weeks, or even months."

Boyer thought it was not possible to effect the reduction after four or six weeks; but Cerpelletti, of Trieste, succeeded after seventy days; Sir Astley Cooper at three months; Malgaigne after three months and twenty-one days. Roux succeeded in the case of a young man twenty-two years of age, whose elbow had been dislocated five months. Blackman, of Cincinnati, informs me that he has reduced a lateral dislocation after five months. Brainard, of Chicago, reduced a dislocated elbow, in a boy of nineteen years, after five months and thirteen days. In this case the Surgeon who had first seen the patient supposed that he had reduced the dislocation. Gorre, Gerdy and Drake, succeeded in four cases after six months; and finally Starch claims to have been successful after two years and one month. To which enumeration Denuce has added seventeen other examples, said to have been reduced at various periods, ranging from one month to one hundred and fifteen days.

Nevertheless, the fact is in the main as stated by Boyer; and if so many cases can be found in which Surgeons have succeeded at a later period they are not probably in the proportion of one to ten as compared with the failures; but the failures have not received the same publicity. Nor indeed have all the severe accidents, such as violent inflammation, suppuration, gangrene, and even death, been faithfully declared.

From the *Science and Art of Surgery*, by John Erichson, we take the following. In speaking of neglected dislocations, this distinguished author says: "The latest period at which reduction should be attempted varies much according to the nature of the dislocation. It may be successfully practised at a much later period in the luxations of the orbicular than of the hinge joints; and it is especially in the shoulder that these late attempts may be advantageously undertaken. According to Sir A. Cooper, however, the latest period at which reduction, even in this articulation, can generally be successfully effected does not exceed three months and eight weeks for the hip; but within this time it may often be safely accomplished."

It is evident, from the above extracts, that our standard authorities, not only do not approve attempts at reduction in neglected dislocations of long standing, but regard such interference as entirely unjustifiable. They admit the numerous successes upon record, but still hold on to the dogma of Boyer and Sir Astley Cooper.

Notwithstanding my high appreciation of the above mentioned authors, still I am firmly of the opinion that in all favorable cases, that is, where there are no complications, as fractures, compound dislocations, extensive injuries of the soft parts, followed by abscesses, caries of the bone, &c., that if presented in a reasonable length of time after the accident, we should attempt the reduction. I will not say in one, four, or eight months, but let the length of time in each individual case be decided upon its own merits. It is evident to all, that age, sex, temperament, the character of the muscular system, the intensity of the inflammation immediately after the accident, and many other considerations, so influence the difficulties in different cases, that a dislocation of the same joint would be easier reduced at four months in one subject than in another, where the surroundings were more unfavorable, after two months.

The frequency and dangers of accidents which sometimes result from attempts at reduction, have, I am confident, been greatly magnified. Within the past fifteen years I have witnessed, both in the hospitals of Europe and America, a number of attempts at the reduction of old dislocations, and have made several myself, but in no instance have I seen any unpleasant results. If the necessary precautions are taken in the selection of cases, as well as the mode of conducting the effort, with the force properly graduated, accidents will be of rare occurrence.

